# Supplementary material for: Tuning direct-written terahertz metadevices with organic mixed ion-electron conductors
Source: Nat Commun. 2024 Nov 7;15:9639. doi: 10.1038/s41467-024-53372-5 (PMC11544203; doi:10.1038/s41467-024-53372-5)
Supplement: Supplementary file 1 — Supplementary Information [file 41467_2024_53372_MOESM1_ESM.pdf]

Supplementary Information

Tuning direct-written terahertz metadevices with organic mixed ion-electron conductors

+Cristiano Bortolotti[1,2], +Federico Grandi[3,4], Matteo Butti[2], Lorenzo Gatto[3], Francesco Modena[2], Christina Kousseff[5], Iain McCulloc[5], Caterina Vozzi[4], Mario Caironi[2], *Eugenio Cinquanta[4], *Giorgio Ernesto Bonacchini[6]

[1] *Dipartimento di Elettronica, Informazione e Bioingegneria, Politecnico di Milano, Milan, Italy*
[2] *Center for Nano Science and Technology, Istituto Italiano di Tecnologia, Milan, Italy*
[3] *Dipartimento di Fisica, Politecnico di Milano, Milan, Italy*
[4] *Istituto di Fotonica e Nanotecnologie, Consiglio Nazionale delle Ricerche, Milan, Italy*
[5] *Department of Chemistry, University of Oxford, Oxford, UK*
[6] *Department of Information Engineering, Università degli Studi di Padova, Padua, Italy*

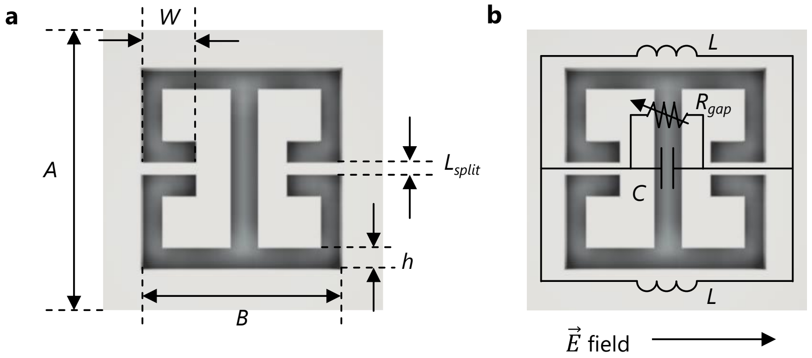

Supplementary Fig. 1. Metaunit design. a, Geometry of the metaunit, where the following parameters were used for the fabrication and EM modeling: A = 50 µm, B = 36 µm, W = 10 µm, h = 4 µm, L_{split} = 2 µm. **b**, Equivalent circuit model for the metaunit, where L corresponds to the self-inductance of the structure, C to the capacitance established by the gap region, and R_{gap} the variable, gate-controlled resistance introduced by the OMIEC. The arrow at the bottom of (**b**) displays the polarization of the electric field, which is orthogonal to the gap.

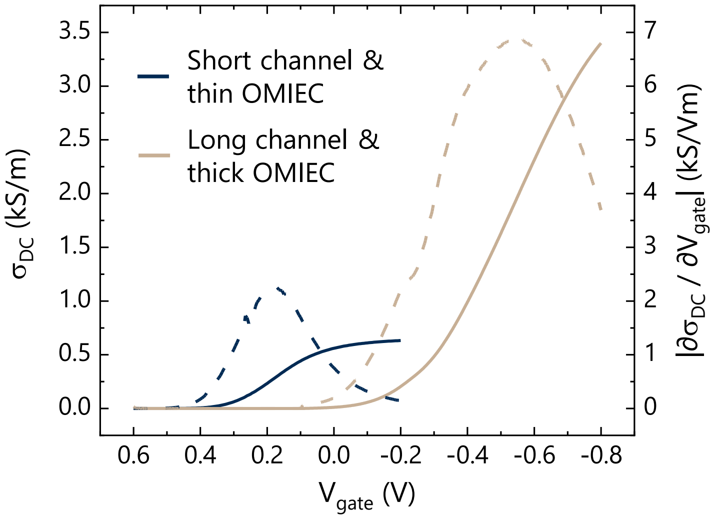

Supplementary Fig. 2. Electrical conductivity measurements. Conductivity obtained from the electrical characterization of two different p(g2T-TT)-based OECTs. The solid blue line corresponds to σ_{DC} of a transistor with W/L = 1500 µm / 3 µm, and with OMIEC thickness of approximately 15 nm. The solid ocra line refers to the data from an OECT with W/L = 3 mm / 2 mm, and with OMIEC thickness of approximately 850 nm. Dashed lines correspond to the derivative of the conductivities (right axis in the plot).

This set of data displays how longer channel lengths and higher OMIEC thickness contribute to the negative shift of σ_{DC}, as well as to its increased steepness with V_{gate}.

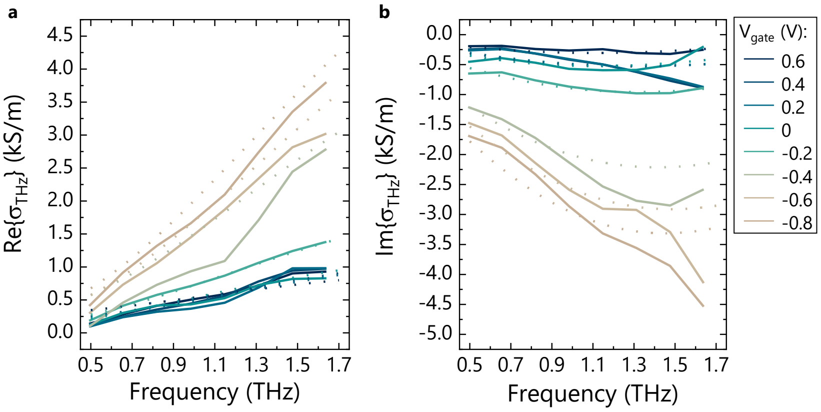

c

V_{gate} [V]	ω_P [THz]	τ [fs]	c_1
0.6	61.6	54	-0.85
0.4	75.4	49	-0.94
0.2	75.4	48	-0.94
0	66.6	57	-0.96
-0.2	96.1	49	-0.97
-0.4	151.4	46	-0.97
-0.6	172.1	44	-0.98
-0.8	184.1	45	-0.98

Supplementary Fig. 3. Drude-Smith model. a,**b** Real (a) and imaginary (b) parts of σ_{THz}, where the dashed lines represent the fit performed with the Drude-Smith model. **c**, Table of the fitting parameters.

a

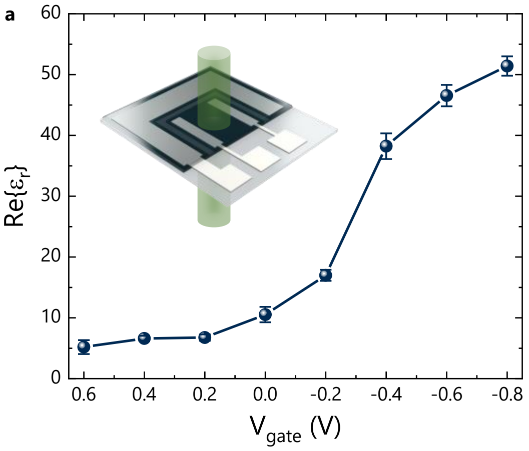

b

V_{gate} [V]	$Re\{\varepsilon_r\}$	$Re\{\sigma_{THz}\}$ | [S/m]
0.6	5.19008	282.27046
0.4	6.6056	252.70206
0.2	6.75357	238.06051
0	10.53029	239.48636
-0.2	17.211675	367.4782245
-0.4	37.524593	458.18735
-0.6	46.545	731.94037
-0.8	51.40917	945.03362

c d

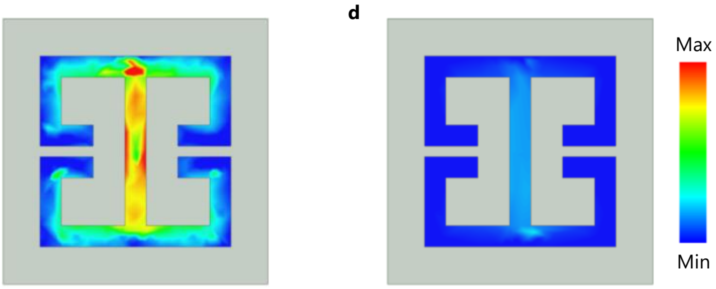

Supplementary Fig. 4. Simulation parameters. a, Graph displaying the voltage dependence of the OMIEC real permittivity of the OMIEC (error bars correspond to the SE with a sample of 5 measurements). **b**, Real permittivity and terahertz conductivity data used for the parametric simulations of the metadevice. **c,d**, Electric field distributions calculated at the resonance frequency for a metadevice with an undoped (**c**, $Re\{\varepsilon_r\}$ = 5.19008 and $Re\{\sigma_{THz}\}$ = 282.27046 S/m) and electrostatically doped OMIEC layer (**d**, $Re\{\varepsilon_r\}$ = 51.40917 and $Re\{\sigma_{THz}\}$ = 945.03362 S/m).

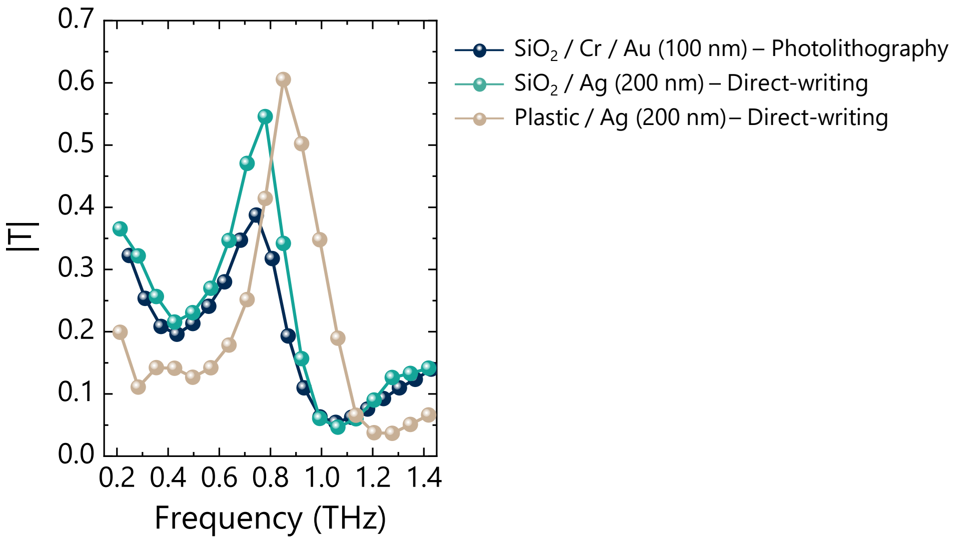

Supplementary Fig. 5. Comparison of fabrication techniques. Transmission spectra of bare metasurfaces (no OMIEC or electrolyte) fabricated with either conventional photolithography (Cr/Au: 5/100 nm on fused silica) and femto-second laser ablation (Ag: 200 nm on fused silica and plastics). The direct-written metasurfaces exhibit higher quality factors likely due to the lower sheet resistance of the thicker Ag layer with respect to the Au one. The device on plastics is characterised by a higher $f_{res,0}$ due to the lower permittivity of the polyimide with respect to fused silica.

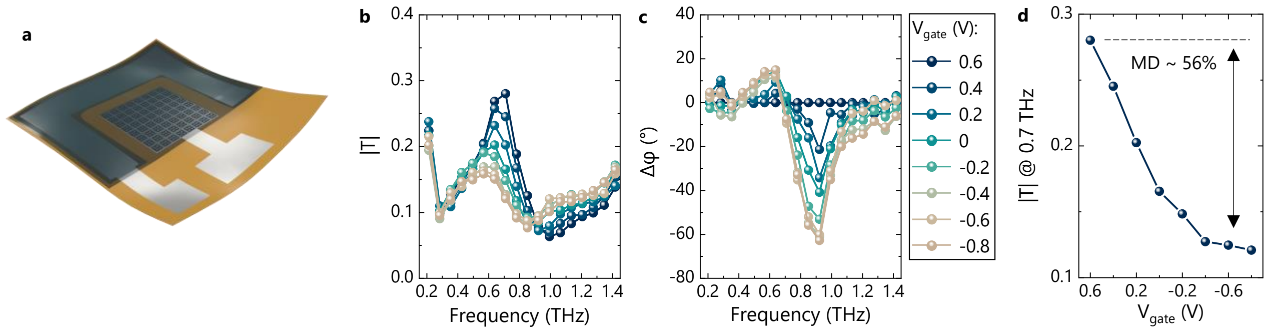

Supplementary Fig. 6. Flexible fully direct-written metadevice. a, Representation of the tunable, fully direct-written complementary metasurface on a flexible polyimide substrate. **b,c** Amplitude and phase modulation of the complex terahertz transmission, where the magnitude (**b**) is calculated with respect to air, while the phase (**c**) uses the 0.6 V spectrum as reference. **d**, Voltage dependence of the transmission at ~0.7 THz.

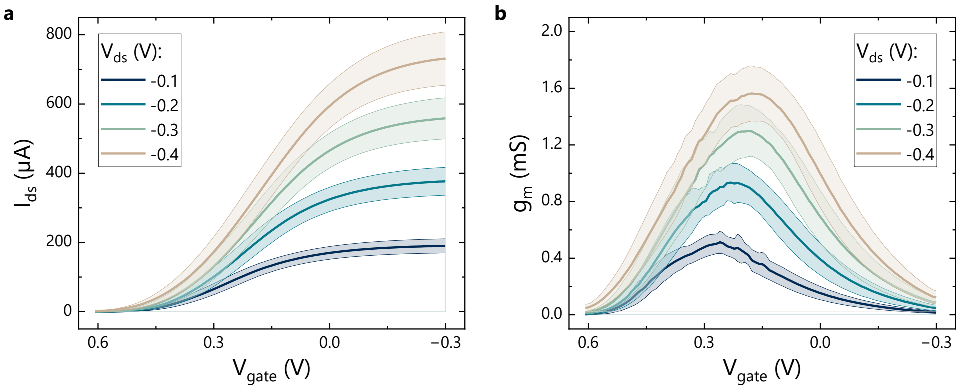

Supplementary Fig. 7. Device reproducibility. The device-to-device reproducibility of the charge modulation performances within the inkjet-printed p(g2T-TT) layer was assessed through the electrical characterization of 5 OMIEC-based transistors, which had the following parameters: Cr/Au source-drain and gate electrodes (2 nm / 30 nm), W/L = 1800 µm / 3 µm, inkjet-printed OMIEC and iongel (as for the metadevices). **a**, The transcharacteristic curve of the transistor yields a maximum Relative Standard Deviation (RSD) in $I_{ds,max}$ of approximately 10% (shaded area corresponds to SD). **b**, For the maximum transconductance $g_{m,max}$, the RSD is comprised between 10% at V_{ds} = -0.4 V and 15% at V_{ds} = -0.1 V.
